# 3-D structural interactions and quantitative structural toxicity studies of tyrosine derivatives intended for safe potent inflammation treatment

**DOI:** 10.1186/s13065-016-0169-9

**Published:** 2016-04-30

**Authors:** Ayarivan Puratchikody, Dharmaraj Sriram, Appavoo Umamaheswari, Navabshan Irfan

**Affiliations:** Drug Discovery and Development Research Group, Department of Pharmaceutical Technology, Anna University Chennai, BIT Campus, Tiruchirappalli, 620024 India; Pharmacy Group, Birla Institute of Technology and Sciences, Pilani, Hyderabad Campus, Jawahar Nagar, Secunderabad, Telangana 500 078 India

**Keywords:** Anti-inflammatory, Tyrosine derivatives, Docking, ADMET descriptors, Osiris

## Abstract

**Background:**

Drugs that inhibit cyclooxygenase-2 (COX-2) while sparing cyclooxygenase-1 (COX-1) represent a new attractive therapeutic development and offer new perspective for further use of COX-2 inhibitors. Intention of this work is to develop safer, selective COX-2 inhibitors that do not produce harmful effects.

**Results:**

A series of 55 tyrosine derivatives were designed for evaluation as selective COX-2 inhibitors and investigated by in silico for their anti-inflammatory activities using C-Docker. The results of docking study showed that 35 molecules were found to selectively inhibit the enzyme COX-2. These molecules formed stable π hydrophobic and additional van der Waals interactions in the active site side pocket of COX-2. The molecules selected from docking studies were examined through ADMET descriptors and Osiris property explorer to find its safety profile as well. The tyrosine derivatives containing toxic fragments were eliminated.

**Conclusion:**

The results conclude that out of 55, 19 molecules possessed best binding energy (< −3.333 kcal/mol) and these molecules had more selective and safer COX-2 inhibitor profile compared to the standard celecoxib.

**Graphical abstract:**
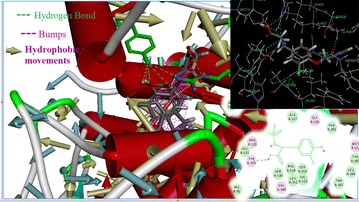
3-D structural interactions of COX-2 inhibiting tyrosine derivatives.

## Background

Cyclooxygenase-1 (COX-1) and Cyclooxygenase-2 (COX-2) are two discrete isoforms of cyclooxygenase enzyme. These enzymes play a catalytic role in transfiguration of arachidonic acid to prostaglandins in the cyclic pathway of arachidonic acid [1, 2]. Prostaglandins (PGs) are involved in various pathophysiological conditions such as inflammation, carcinogenesis, cardiovascular activity etc. Generally, COX-2 is not detectable in most normal tissues, but it is induced by pro-inflammatory cytokines, growth factors and carcinogens. This fact indicates the role of COX-2 in inflammation [3]. Rheumatoid synovium expression of COX-2 is up regulated in inflammatory tissues resulting in the production of prostaglandin precursors which ultimately gets converted into PGs [4].

Some of the coxib derivatives, Rofecoxib, Celecoxib, Etoricoxib and Valdecoxib are selective COX-2 inhibitors that act by blocking COX-2 enzyme responsible for inflammation and pain [5]. Most of these coxib derivatives have been voluntarily withdrawn from the worldwide market due to safety concerns of an increased risk of cardiovascular events in patients. Due to greater therapeutic effect, Celecoxib is remaining in the market, even though it have a risk of serious and potentially fatal adverse cardiovascular thrombotic events, myocardial infarction and stroke [6].

Importantly, design of agents with higher anti-inflammatory potential and less side effects is one of the most challenging areas in the inflammation. On review of literature, researchers have proved anti-inflammatory effects for dibromotyrosine derivatives [7]. In this concern, we searched for tyrosine scaffold from the natural sources since the biologically active natural compounds are composed of very complex structures. This complexity makes the compounds extremely novel. The marine sponges such as *Psammaplysilla purpurea* and *Ianthella basta* are known to produce biogenetically related bromotyrosine derived secondary metabolites [8, 9]. These observations prompted us to design and develop analogue(s) of bromotyrosine derivatives which specifically inhibits COX-2 with improved biological activity. As part of this drug development, an effort has been made to develop higher-quality drug candidates through computational techniques.

## Methods

### Ligand preparation

**Fig. 1:**
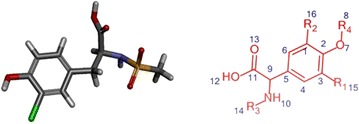
3D and 2D structure of energy minimized tyrosine derivatives

**Fig. 2:**
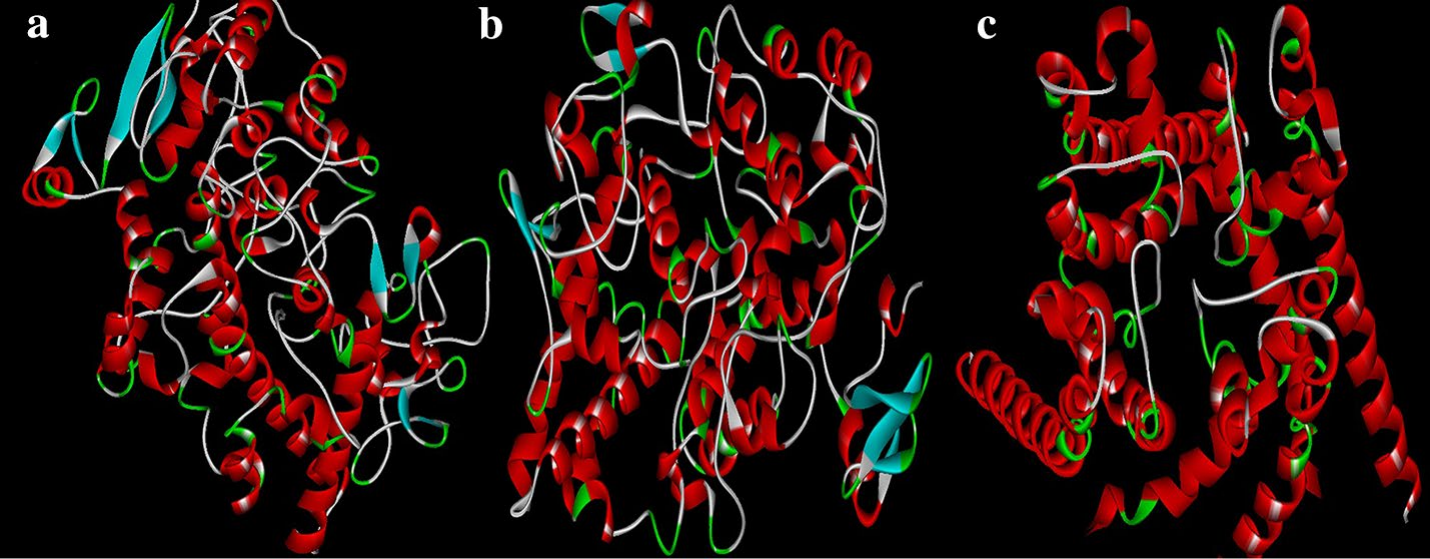
Minimized secondary structure of **a** COX-2 (3NT1) **b** COX-1 (3KK6) **c** hERG protein (homology model)

A library of novel 55 tyrosine molecules were designed based on the SAR studies of known anti-inflammatory drugs. These molecules were generated with tyrosine as a basic skeleton. The 15 (R_1_) and 16 (R_2_) position of aromatic ring hydrogen was substituted with different electronegative groups such us, –I, –Br, –Cl and –NO_2_. Further, one hydrogen atom of –NH_2_ group in 14 (R_3_) position was replaced by –SO_2_CH_3_ group. The eighth position (R_4_) of phenolic –OH group hydrogen was replaced by diverse heterocyclic fragments (Fig. 1). The structures of these molecules were drawn in Hyperchem molecular modeling and visualization tool (version 7.5) and the energies were minimized using ADS. The minimized ligands and proteins were saved in structure data (.sd) and.pdb format (Fig. 2) respectively for further studies.

### Docking study

**Fig. 3:**
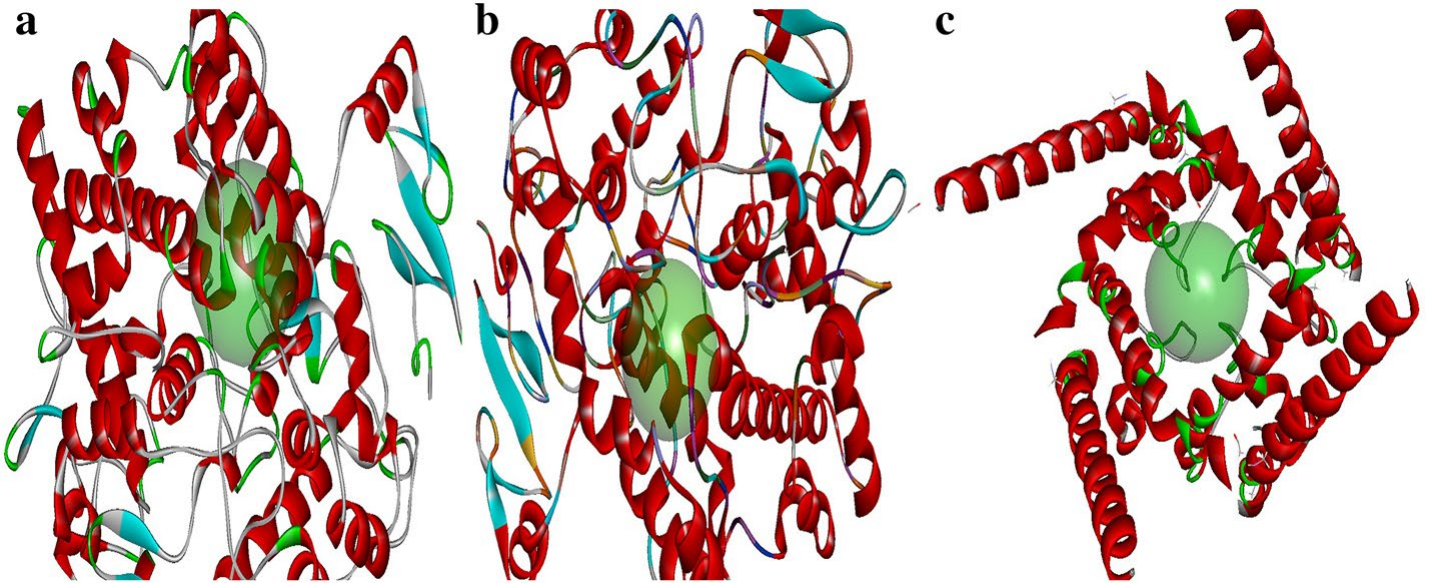
Binding site representation of proteins **a** COX-2 **b** COX-1 **c** hERG_IFD_S- terfenadine_model_1

The docking study was performed using Accelyrs Discovery Studio client version 2.5 software (Accelyrs Inc., http://www.accelrys.com). The X-ray crystallographic structure of COX-2 (PDB ID 3NT1) protein bound with naproxen was acquired from the protein data bank (PDB) at a resolution of 1.73 Å (Table 1). The active site was defined with a 8.500 (Å) radius around the bound inhibitor which covered all the active site amino acids of the COX-2 protein. A grid-based molecular docking method, C-DOCKER algorithm was used to dock the small molecules into the protein active site. The designed structures were submitted to CHARMm (Chemistry at HARvard Macromolecular Mechanics) force field for structure refinement. All water molecules, bound inhibitor and other hetero atoms were removed from the macromolecule and polar hydrogen atoms were added. The designed structures were also verified for its valency, missing hydrogen and any structural disorders like connectivity and bond orders. Energy minimization was carried out for all compounds using CHARMm force field to make stable conformation of protein with an energy gradient of 0.01 kcal/mol/A°. A final minimization of the ligand in the rigid receptor using non-softened potential was performed. For each final pose, the CHARMm energy (interaction energy plus ligand strain) and the interaction energy alone were calculated. The poses were sorted by CHARMm energy and the top scoring (most negative, thus favorable to binding) poses. The energy minimized individual proteins and the designed structures along with the binding site sphere radius (Table 2; Fig. 3) and the X, Y and Z coordinates (Table 3) were submitted to the C-Docker job parameter. The docked conformation which had the lowest C-Docker energy was selected to analyze the mode of binding pattern. The C-Docker energy score, hydrogen bond and VDW interactions were visualized in C-Docker report and used for further analysis.

**Table 1:** Protein resolution and its stable conformational energy

PDB ID	Description	Resolution (Å)	Initial potential energy (kcal/mol)	Final potential energy (kcal/mol)
3NT1	High resolution structure of naproxen:COX-2 complex	1.73	−492,721	−500,025
3KK6	Crystal structure of COX-1 in complex with celecoxib	2.75	248,964,312.95	−34,200.97
HM^a^	hERG IFD S terfenadine model 1	–	−15,609	−21,445.6

^a^ Homology modeling

**Table 2:** Binding sphere radius and X, Y and Z coordinate values of defined protein binding site

Protein PDB ID	Binding sphere radius (Å)	Coordinates (Å)		
		X	Y	Z
3NT1	8.50067	−40.406	−51.829	−22.502
3KK6	6.98804	−32.413	−51.829	−5.617
hERG_IFD_S−terfenadine_model_1	7.41161	189.526	−0.442	40.737

**Table 3:** C-Docker docking protocol parameters

Parameters	Inputs
Input receptor	../Input/3NT1.dsv
Input ligands	C:\Users\g\Desktop\all 55 new.sd
Input site sphere	−40.4058, −51.8288, −22.5019, 8.50067
Top hits	1
Random conformations	10
Random conformations dynamics steps	1000
Random conformations dynamics target temperature	1000
Include electrostatic interactions	True
Orientations to refine	10
Maximum bad orientations	800
Orientation VDW energy threshold	300
Simulated annealing	True
Heating steps	2000
Heating target temperature	700
Cooling steps	5000
Cooling target temperature	300
Force field	CHARMm
Use full potential	TRUE
Grid extension	8
Ligand partial charge method	CHARMm
Random number seed	314,159
Final minimization	Full potential
Random dynamics time step	0.002

The potential fatal adverse effects viz ulcerogenecity and cardiotoxicity were determined by C-Docker using the crystal structures of COX-1 in complex with celecoxib (3KK6:2.75 Å) and hERG_IFD_S-terfenadine_model_1 [Homology model (HM)] (Table 1) which were chosen from the PDB and Schrodinger website respectively. The binding sites of the COX-1 (3KK6) and hERG proteins were defined with the radii of 6.988 and 7.411 Ǻ respectively. The novelty of the final hits was confirmed using SciFinder [10] and PubChem [11] structure search tools.

### Docking protocol validation

The validation of the docking protocol is essential to analyse the prediction ability of the proposed method [12]. In this study, validation is performed by two methods to verify whether our docking protocol is able to discriminate selective and non-selective COX-2 inhibitors. To start with, four native co-crystallised ligands of selective and non-selective COX-2 inhibitors were identified and kept as reference template. The structures of these ligands were drawn separately and its energies were minimized. RMSD values were calculated and analysed by redocking the energy minimised ligand on reference template by molecular overlay technique in ADS. In the second method, the structures of various selective and non-selective inhibitors were drawn and the potential energies of the molecules were minimized with the help of conjugated gradient algorithm. Further, these molecules were docked with the COX-2 (3NT1) protein to calculate the binding energies. The experimental IC_50_ activity values of these molecules were compared with its corresponding predicted C-Docker energy values and the point plot is graphed to identify the correlation between the IC_50_ and C-Docker energy.

### Toxicity study

#### ADMET descriptors

Most of the failure of drug candidates during clinical trials is due to its poor pharmacokinetic and toxicity properties [13]. Hence, prediction of ADMET properties prior to expensive experimental procedures is considered to be essential for the selection of successful candidates. In this work, in silico ADMET studies were done using ADMET descriptors algorithm of ADS. This protocol uses the six pharmacokinetic parameters like Human Intestinal Absorption (HIA), Blood–Brain–Barrier (BBB) penetration, aqueous solubility, hepatotoxicity levels, cytochrome P450 2D6 inhibition and Plasma Protein Binding (PPB) to quantitatively predict the molecular properties of selected 35 ligands.

#### Osiris property explorer

Toxicity risks (mutagenicity, tumorigenicity, skin irritation, reproduction) and physicochemical properties (drug likeness and drug score) of the selected 35 tyrosine derivatives were calculated using OSIRIS Property Explorer (free web-based program). The drug likeness (d) was calculated with the following equation by summing up the scores of molecular fragments (V_i_) and n indicates the number of molecular fragments [14].

1 $${\text{d}} = \frac{{\sum {\text{v}}_{\text{i}} }}{{\sqrt {\text{n}} }}.$$

The fragment list was created by shredding 3300 traded drug as well as 1500 commercially available chemicals.

The drug score (ds) combines drug-likeness, cLogP, logS, molecular weight and toxicity risks in one handy value that may be used to judge the compound’s overall potential to be qualified as a drug. This value was calculated by multiplying the contributions of individual properties with Eq. (1) [15].

2 $${\text{ds}} = \uppi \left( {\frac{1}{2} + \frac{1}{2}{\text{si}}} \right)\cdot\uppi {\text{ti}}$$

ds is the drug score. s_i_ are the contributions calculated directly from of cLogP, logS, molecular weight and drug-likeness t_i_ is the contribution taken from the four toxicity risk types via the Eq. (2) which describes a spline curve.

## Results and discussion

### Docking

The results of C-Docker protocol run were analysed. These results have provided essential information relating to the orientation of the tyrosine derivatives in the active site of proteins (3NT1, 3KK6, hERG).

### Molecular docking

**Fig. 4:**
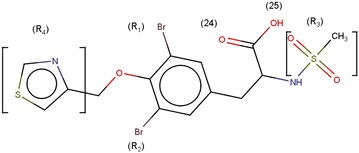
Structure of molecule **8**

**Fig. 5:**
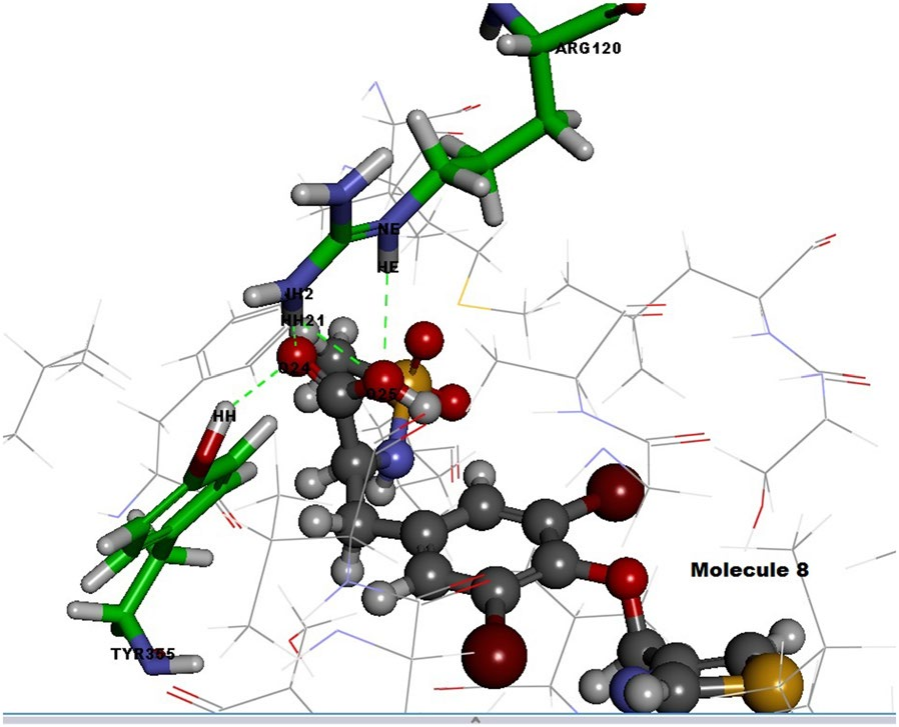
Interactions of molecule **8** with active site amino acids of COX-2 protein

**Fig. 6:**
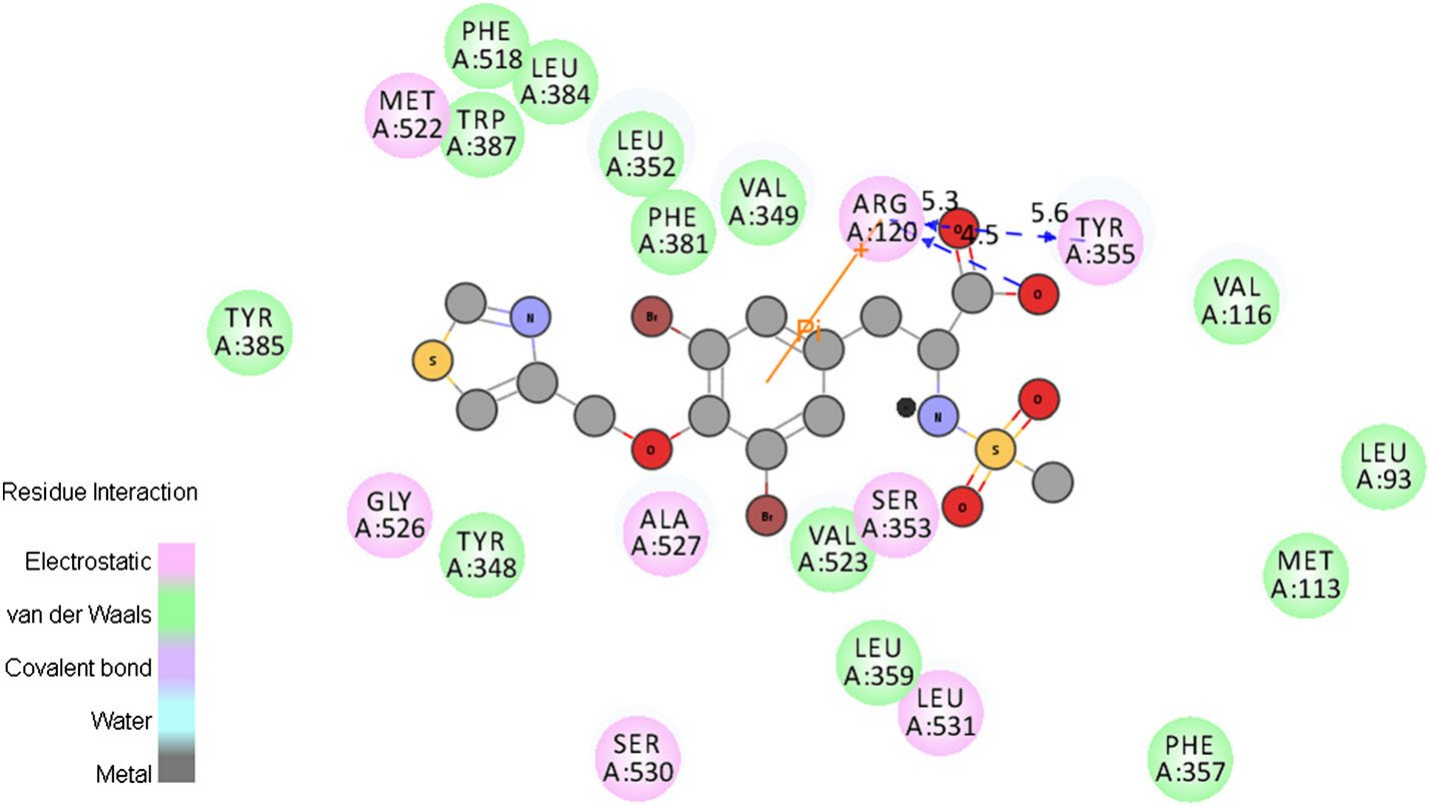
2D Interactions view of molecule **8** with active site amino acids of COX-2 protein

In this study, 35 drug-like hit compounds were selected from the designed 55 tyrosine derivatives based on their better binding affinity (–C-Docker energy) compared to the standard celecoxib (Table 4). The active site was defined based on the bound inhibitor, naproxen, in a crystal structure of COX-2 (PDB code 3NT1). The important criteria considered in the selection of best hit compounds was binding modes, molecular interactions with the active site components and fitness scores. Evaluation of the interaction pattern of tyrosine derivatives makes clear that the molecule **8** (Fig. 4**)** have six folds higher affinity (−78.7003) in the COX-2 active site compared to standard celecoxib (17.3339). This interaction affinity is due to the 24th oxygen atom of the carboxylic group in tyrosine moiety has formed two site point interactions with the binding site residue of Arg^120^ and Tyr^355^ residue. The 25th oxygen atom of the molecule produced one ligand point interaction with Arg^120^ residue which allows major interaction impact of the tyrosine derivatives on catalytic domain of COX-2 protein. Besides, aromatic ring of the tyrosine skeleton make π-cationic interaction with Arg^120^. This created a stable conformation of the molecule **8** in the hydrophobic binding site of the COX-2 protein. This long hydrophobic channel creates cyclooxygenase active site that inhibits the inflammation via non-steroidal anti-inflammatory drugs. This active site lengthen from the membrane binding domain to the region where the catalyzed chemical reaction takes place [16, 17]. In addition, R_1_ and R_2_ bromine substitution had generated VDW interaction with Val^523^ and Phe^518^ that permitted the molecule **8** to access an additional side pocket which is a pre-requisite for COX-2 drug selectivity. This structural modification may be attributed to the interchange of valine at position of 523 in COX-2 for a relatively bulky isoleucine residue in COX-1 [5]. The substitution of 1, 3-thiazole ring at –OH (R_4_) position of tyrosine induced the VDW and electrostatic interactions with the active site amino acids. It created conducive chemical environment in the COX-2 binding site. Substitution of electronegative sulfonyl group at R_3_ position enhanced the binding potential of the molecule by interacting with Ser^353^ (Figs. 5, 6). It is confirmed from this study that the COX-2 selectivity of the molecule **8** is higher than the standard celecoxib. The rest of 34 molecules were examined and found to have more stability when compared to the standard.

**Table 4:** Interaction energy values of tyrosine derivatives and celecoxib with COX-2 protein

Name	C-Docker energy^a^	–C-Docker interaction energy^a^	Initial potential energy^a^	Initial RMS gradient	Electrostatic energy^a^	Potential energy^a^	VDW energy^a^	RMS gradient
Molecule_8	−78.7003	4.96727	−74.2658	16.3263	−199.774	−155.629	3.78158	0.09694
Molecule_54	−46.1094	3.80434	9.73689	40.9916	−161.106	−129.460	5.45173	0.09737
Molecule_23	−45.4158	1.08668	−2.98987	44.0659	−177.976	−139.880	5.17214	0.09761
Molecule_6	−40.1233	1.50834	339.920	91.3010	−131.124	−106.360	1.62197	0.09667
Molecule_14	−38.0308	9.72515	−93.3437	6.78104	−128.448	−98.3557	2.48238	0.08123
Molecule_50	−32.9449	−3.8949	25.7593	47.7124	−133.414	−112.472	−0.05903	0.08110
Molecule_25	−29.4798	14.5849	14.0717	43.5888	−142.506	−118.107	1.45489	0.07719
Molecule_51	−28.5191	0.90861	32.0508	46.2969	−130.255	−100.616	2.08610	0.08137
Molecule_24	−28.4505	16.3299	534.240	568.860	−140.619	−104.274	3.04089	0.09149
Molecule_11	−26.1386	6.71301	61.7373	44.7827	−151.439	−136.499	−1.89433	0.09066
Molecule_10	−23.4787	21.0787	71.8921	63.5300	−157.857	−126.759	6.24669	0.09716
Molecule_20	−21.3714	17.7833	−17.3987	40.1040	−120.253	−99.3152	4.00568	0.09156
Molecule_21	−20.4346	21.4014	−55.1410	4.32287	−79.2812	−58.7042	1.69521	0.08615
Molecule_57	−15.0159	13.1286	28.7444	53.0095	−162.501	−128.173	8.40306	0.09806
Molecule_58	−12.0458	3.82902	−56.6613	20.5821	−152.031	−129.655	3.88633	0.09311
Molecule_7	−5.28412	22.9306	55,568.4	75,666.6	−150.99	−121.105	1.02490	0.09610
Molecule_67	−3.39829	11.7573	26.7832	42.2915	−123.539	−103.737	2.94580	0.09285
Molecule_59	−1.19358	14.2210	−89.0706	14.9038	−132.811	−104.716	3.23357	0.08664
Molecule_13	0.274143	19.9477	73.5331	48.1590	−130.355	−111.012	0.14158	0.09153
Molecule_17	0.957257	13.0669	−13.8560	6.29743	−42.2671	−25.9434	−1.71694	0.08943
Molecule_15	0.961175	18.3100	−37.7612	6.14656	−75.5862	−50.066	3.31430	0.08757
Molecule_102	4.763580	38.6705	−1.47768	6.52181	−20.0264	−13.5243	−6.95490	0.08871
Molecule_52	7.997040	16.8484	487.293	105.229	−155.493	−119.589	−0.16776	0.09978
Molecule_26	8.272020	21.8179	42.0749	38.5993	−138.631	−94.2976	2.46568	0.08678
Molecule_146	8.494660	38.3012	13.8468	6.52572	−12.4255	−7.32723	−7.01223	0.09203
Molecule_103	9.218700	41.1444	−0.67757	6.08668	−21.515	−13.0472	−4.12813	0.09382
Molecule_12	9.37307	23.8593	599.169	124.448	−133.207	−95.3196	−0.06212	0.09771
Molecule_99	10.0093	38.4689	10.9431	4.96463	−5.95502	0.97673	−4.40147	0.09965
Molecule_154	10.8974	42.4735	10.9119	7.15111	−16.5170	−5.14193	−5.71648	0.86146
Molecule_9	11.5098	24.9470	65.3320	46.7416	−120.920	−71.2045	2.39190	0.09569
Molecule_113	12.1402	41.6382	28,289.50	39,402.2	−136.929	−83.7555	2.70303	0.09185
Molecule_60	12.5198	19.1621	−13.2592	6.10851	−41.6039	−25.2553	−0.89500	0.08769
Molecule_115	12.5673	46.3928	−11.8405	6.12586	−44.0638	−23.2031	1.68847	0.09377
Molecule_141	12.8093	32.4320	−5.41508	4.20617	−29.7202	−17.6498	−0.35700	0.09705
Molecule_117	17.0983	38.6338	2021.79	2299.10	59.3405	96.7284	3.50862	0.09529
Celecoxib	17.33395	33.9253	13.8933	42.5446	−139.661	−117.986	6.21732	0.09936
Molecule_142	17.3898	38.1553	−79.2244	15.0265	−128.565	−93.4084	2.66973	0.09101
Molecule_100	17.9025	29.9299	6222.05	6373.98	−160.241	−112.801	1.91017	0.08234
Molecule_105	17.9411	39.9044	3.14311	5.19254	−19.9248	−12.5248	−3.32516	0.09908
Molecule_110	18.7239	44.9821	6.43866	5.82078	−15.4753	−6.75722	−1.88785	0.09904
Molecule_107	20.4115	36.5229	18.9587	5.76753	−11.5264	0.057840	−2.43628	0.09055
Molecule_98	20.7154	30.5548	−1.67995	5.36936	−21.3184	−9.82350	−1.23215	0.09869
Molecule_104	23.4169	41.1073	6.86145	4.83158	−23.4274	−2.71648	−5.74037	0.0969
Molecule_114	24.2130	49.8248	5.38092	5.21023	−12.5330	−4.10493	−4.12701	0.08961
Molecule_101	24.4073	38.7888	9.88176	16.3253	−17.7871	−5.53674	−3.82927	0.08234
Molecule_143	25.1057	38.8955	4.73240	5.43985	−23.7807	−9.70408	−2.10607	0.08908
Molecule_159	25.8484	38.2027	6.37157	5.98037	−34.4232	−3.90721	−5.13228	0.09613
Molecule_122	26.6459	39.9598	2.21425	6.49133	−34.9642	−14.2581	−6.04252	0.08389
Molecule_111	29.4514	42.7783	38.9483	46.4718	−127.864	−82.6885	3.20745	0.08620
Molecule_118	30.2871	43.3848	13.8351	7.06563	−23.0209	−1.49548	−7.16945	0.08975
Molecule_144	31.0438	41.6663	16.4609	6.42745	−18.8412	4.78885	−6.92729	0.09901
Molecule_150	34.7730	46.4684	36.3206	7.23252	−7.94199	11.2902	−2.19893	0.08886
Molecule_112	35.1376	46.2887	8.39953	6.33198	−33.0042	−4.69838	−5.58198	0.09233
Molecule_151	35.3649	45.4588	33.7593	7.10073	−14.0113	18.3148	−3.51091	0.08832
Molecule_152	41.9392	45.3560	90.9266	30.9076	−12.7073	16.6751	−3.82475	0.09296

^a^The energies of the molecules are indicated in kcal/mol unit

The COX-2 selectivity of the 55 tyrosine derivatives was compared with COX-1 enzyme. In this COX-1 docking study, the designed molecule had not created appropriate conformation inside the active site of COX-1 enzyme due to the bulky amino acid residue Ilu^523^ and non-polar moieties of the His^513^. The VDW space of the tyrosine molecules in COX-1 chemical space of the active site is in conflict with the receptor essential volume. This conflict creates steric repulsion between side chain amino acids of the COX-1 and designed molecules. It strongly evidenced that there is a large decrease in the affinity of the designed tyrosine derivatives with COX-1 when compared to the celecoxib. The above results proved that the tyrosine derivatives are more selective on COX-2 than COX-1.

### Ulcerogenic interaction

**Fig. 7:**
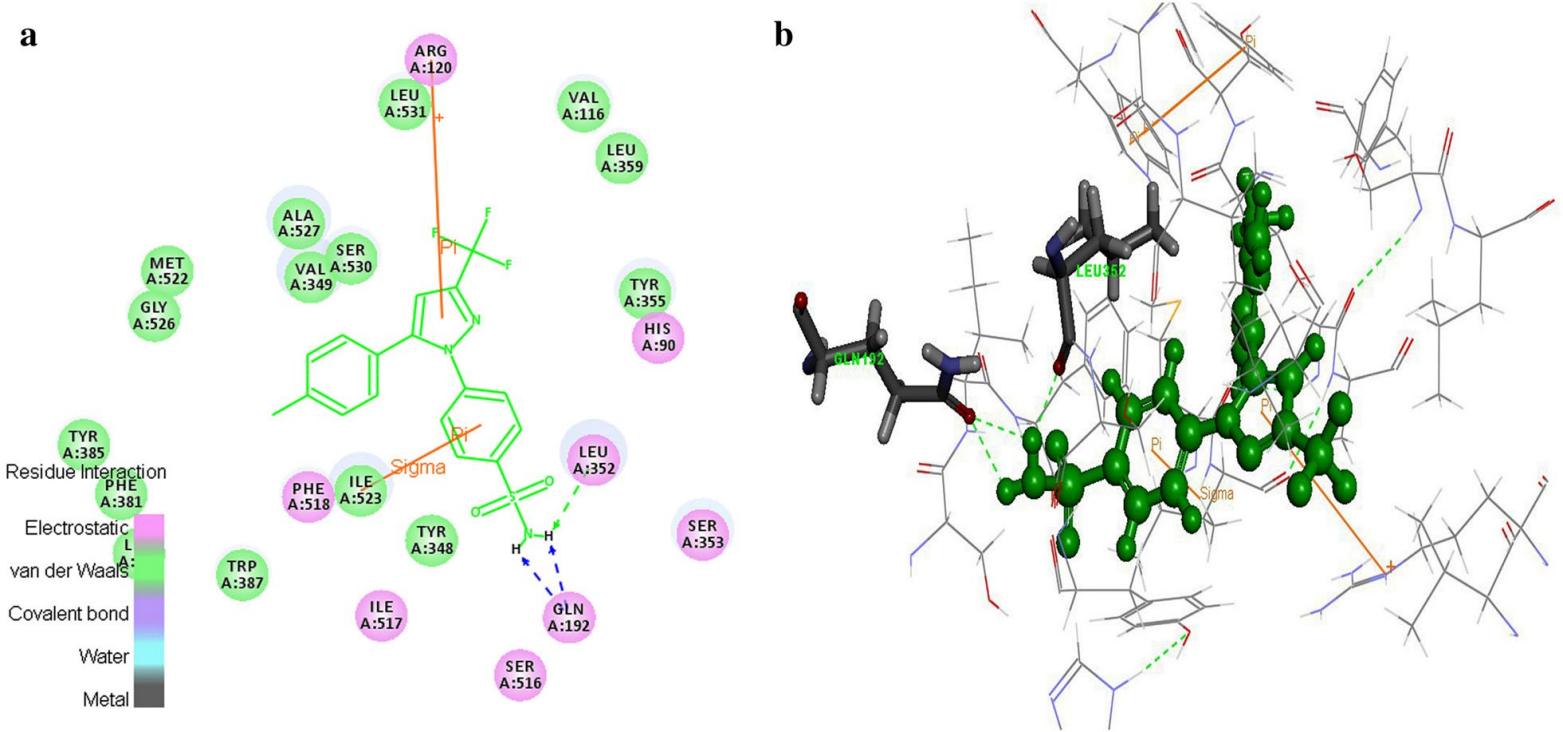
Celecoxib interaction map with the COX-1 protein **a** 2D view of non-bonded interactions **b** 3D interaction view

**Fig. 8:**
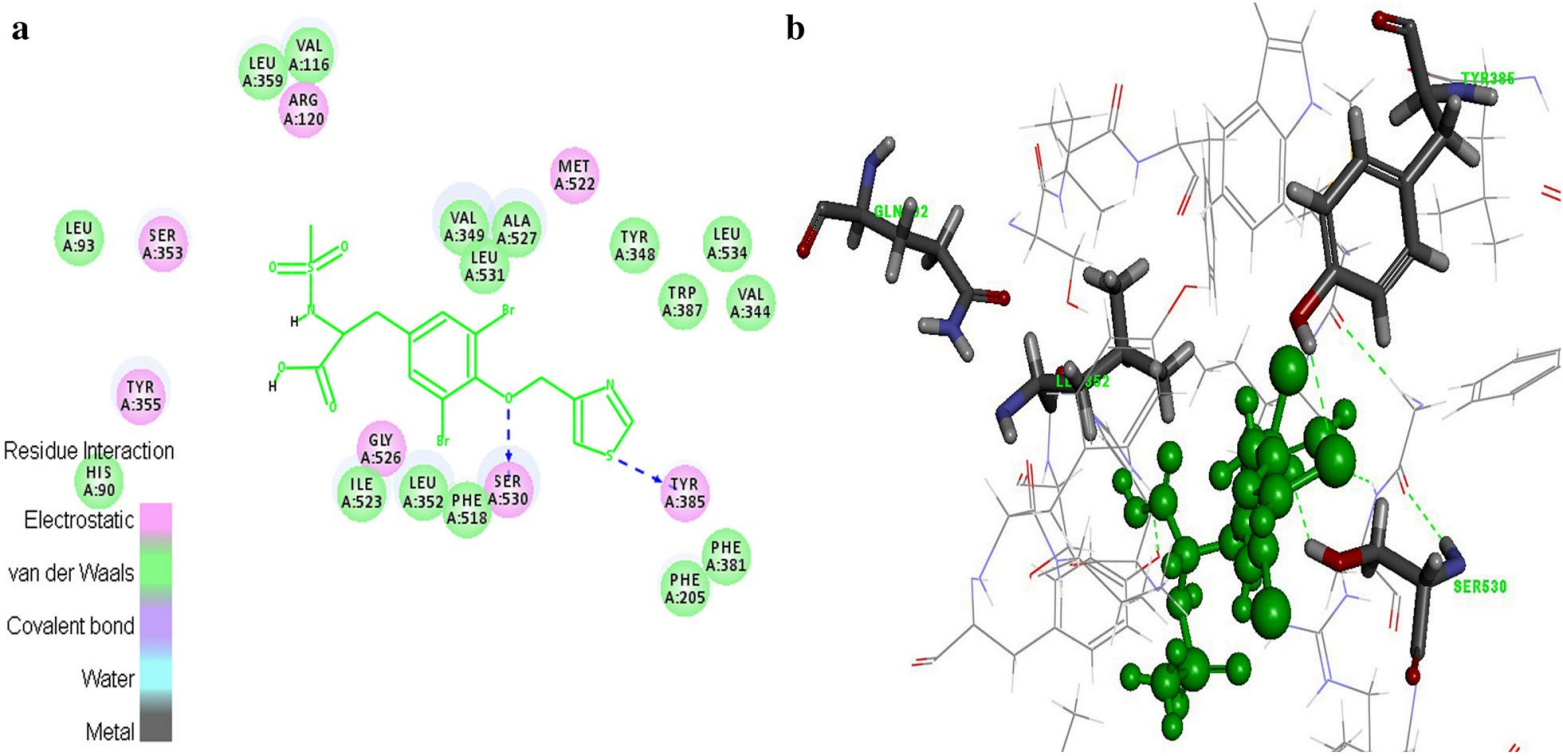
Tyrosine derivatives interaction map with the COX-1 protein **a** 2D view of non-bonded interactions **b** 3D interaction view of hERG protein interaction

The enzyme COX-1 played pivotal role in the maintenance of mucosal integrity in the gastrointestinal tract. It is believed that the ulcerogenic effects of non-steroidal anti-inflammatory drugs is owing to exclusive inhibition of COX-1 [18]. The interaction between the designed 55 tyrosine moiety and COX-1 protein aided to identify the ulcerogenicity level of designed molecules. The results of docking studies (C-Docker) revealed that the designed tyrosine derivatives exhibited more binding energy which was in contrast with the standard celecoxib (Table 5). The standard drug formed, one sigma-π, π-cationic and two hydrogen bond interaction with the Ile^523^, Arg^120^, Gln^192^ and Lue^352^ amino acids respectively (Fig. 7). These bonds support the celecoxib to fit into the cavity of COX-1 enzyme. On the other hand, the designed tyrosine derivatives formed hydrogen bonds with the Tyr^385^ and Ser^530^ (Fig. 8) and there is no other additional interaction with the active site amino acids of COX-1 receptor. Also, the electro negative groups (-Br, -I) of the designed molecules forms intermolecular bumps which disfavors the binding capability of the molecules. These unstable conformations of the designed molecule prove their negligible ulcerogenic side effect.

**Table 5:** C-Docker values for the tyrosine derivatives with COX-1 and hERG protein

Name of the molecule	COX-1		hERG	
	C-Docker energy	–C-Docker interaction energy	C-Docker energy	–C-Docker interaction energy
Molecule_11	11.1931	39.4964	25.6376	36.6014
Molecule_7	15.4566	35.6748	25.7715	38.8912
Molecule_102	18.612	45.7385	6.12591	32.7374
Molecule_99	22.7057	45.0127	13.8034	33.4137
Molecule_10	23.7368	49.2067	17.2666	37.8672
Molecule_113	25.291	51.6406	11.4752	37.2134
Molecule_14	25.5442	45.5213	27.0423	39.9337
Molecule_50	27.2685	42.4608	37.1187	35.2034
Molecule_54	27.9592	45.2948	30.1138	37.0948
Molecule_154	28.0068	48.3108	22.5487	37.1437
Molecule_103	28.5622	49.3350	12.6594	33.7937
Molecule_23	28.9051	51.4072	27.8294	36.9222
Molecule_146	29.7938	48.7906	12.7369	29.7554
Molecule_117	32.24	50.9400	17.502	35.4744
Molecule_122	32.4296	41.5757	23.1628	31.3608
Molecule_21	32.7128	54.7328	26.6071	40.1819
Molecule_8	33.0627	39.4668	36.4622	33.5932
Molecule_115	33.5042	53.2589	16.3142	35.3354
Molecule_105	33.9553	48.2822	20.7848	31.2565
Molecule_114	34.2029	54.9272	19.9584	37.415
Molecule_25	34.4117	51.3171	32.1083	41.2442
Molecule_100	34.7976	46.4333	21.2323	30.1093
Molecule_110	34.9249	53.1217	16.7253	34.8426
Molecule_6	35.033	45.8393	41.2549	36.2332
Molecule_26	35.2188	44.5275	43.3746	38.2527
Molecule_51	35.9835	42.5881	41.6024	38.3912
Molecule_107	36.0181	46.6087	21.6437	29.0359
Molecule_159	36.4927	43.0306	25.964	32.5955
Molecule_142	37.3162	49.1502	22.6982	30.9975
Molecule_141	37.8732	49.0256	23.9516	32.7221
Molecule_15	37.9714	43.6680	37.2577	34.7344
Molecule_58	38.0398	42.8592	43.586	36.3985
Molecule_13	39.4551	49.4952	35.9365	38.5156
Molecule_52	41.2608	41.016	48.0603	37.6181
Molecule_67	41.3861	43.4004	40.4953	37.1043
Molecule_59	41.5117	49.588	51.4686	44.477
Molecule_104	41.5637	48.3222	25.0942	32.4227
Molecule_101	42.6379	48.1987	25.8003	32.6898
Molecule_98	42.9202	48.7329	27.3695	33.4591
Molecule_143	43.1506	48.7602	26.4997	32.3527
Molecule_9	43.4413	49.4292	40.1671	35.6967
Molecule_20	44.4891	48.6384	45.1393	37.1212
Molecule_24	45.1278	54.9812	39.8631	37.5571
Molecule_12	45.21	49.8787	41.6676	37.8414
Molecule_111	45.9116	51.7703	27.1743	33.3161
Molecule_144	46.9694	46.4174	34.4717	35.0657
Molecule_112	47.1361	50.3979	32.8243	35.6348
Molecule_151	48.0494	53.3779	35.1662	37.0065
Molecule_150	48.0592	53.4856	29.3814	36.5024
Molecule_118	48.319	52.7029	29.5539	33.4975
Molecule_17	48.6628	48.8614	45.811	38.6698
Molecule_60	49.6967	49.4645	56.8447	44.3316
Molecule_152	52.4378	51.2126	40.7779	35.7427
Celecoxib	19.4457	51.7111	−0.642396	30.7255

### hERG protein interaction studies

**Fig. 9:**
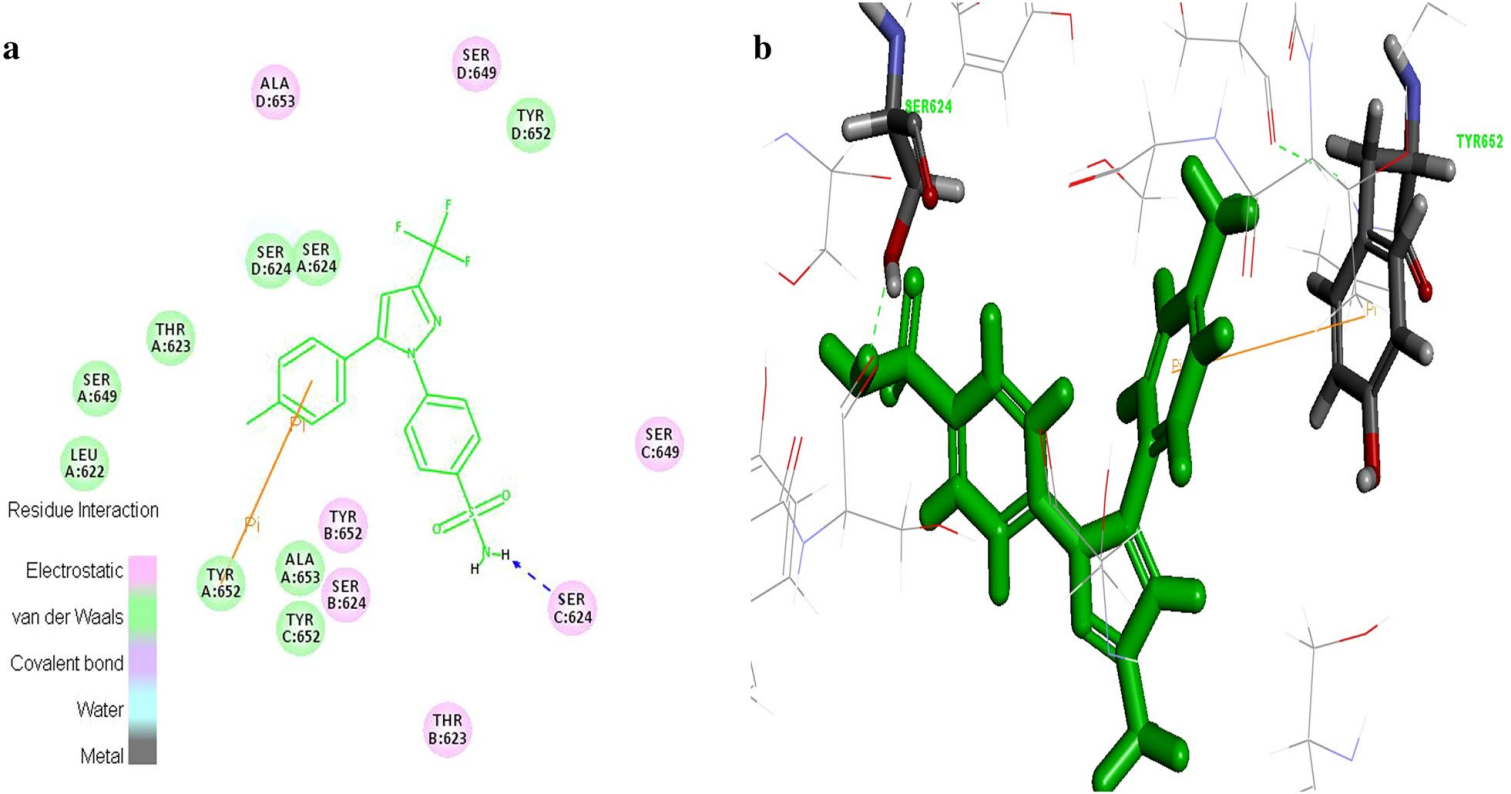
Standard celecoxib interaction map with the hERG protein **a** 2D view of non-bonded interactions **b** 3D interaction view

**Fig. 10:**
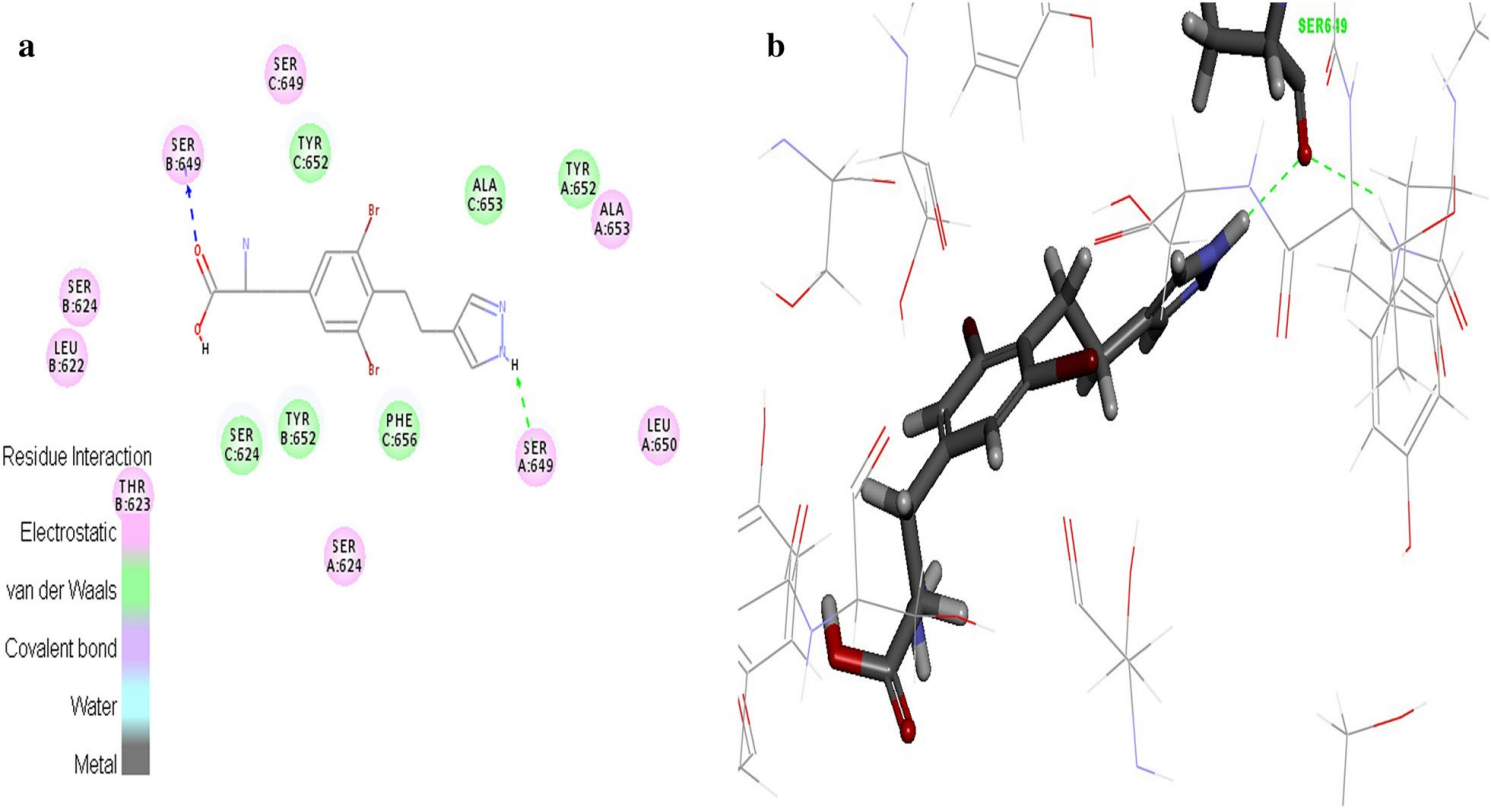
Tyrosine derivatives interaction map with the hERG protein **a** 2D view of non-bonded interactions **b** 3D interaction view

The hERG is the most critical channel involved in drug induced Torsade de Pointes (TdP) arrhythmias. Extra cellular application of celecoxib causes rapid suppression of hERG channels which induces the cardiac disturbances [19]. Evaluation of spatial orientation of the designed molecule interactions with the hERG protein recognizes the cardiotoxicity level of molecules [20]. The results of docking studies indicated that among the 55 designed molecules, 52 molecules possessed more interaction energy against the standard (Table 5). It revealed that these molecules are having less binding affinity to the active site residues of the hERG protein. In standard celecoxib, the benzyl ring creates π-π interaction with the Tyr^652^ (Fig. 9). This enables the celecoxib to fit well into the hydrophobic pocket of COX-2 protein. On contrary, tyrosine derivatives did not form any π-π interactions and the extra volume of the electronegative group substitutions in the R1 and R2 positions which repulse the molecules to bind in the active site (Fig. 10). Hence, the cardiotoxicity of the designed molecules were less when compared to the celecoxib. The selected 35 tyrosine molecules demonstrated high COX-2 selectivity, less COX-1 (ulcerogenic) and hERG (cardiotoxicity) binding affinity. Further, these molecules were examined by ADMET descriptors calculation and OSIRIS properties explorer.

### Docking protocol validation

The results of RMSD values of redocked native co-crystallized ligand of each PDB entry revealed that native ligand conformations including 3NT1 and best docked ligand conformation exactly binds in the experimental protein binding mode. In the docking study performed by first method, RMSD values of best docked conformations ranged from 0.8436 to 1.7674 Å. According to validation protocol, RMSD values of best docked conformation should be ≤2.0 Å [21]. It represents that this docking protocol is able to find an appropriate binding mode. The designed 55 molecules were redocked into the active site of the COX-2 (3NT1) receptor and confirms that these docked molecules followed the similar binding method as in native co-crystallised ligand (Table 6).

**Table 6:** Native co-crystallised ligands and its respective PDB ID with its redocked RMSD values

Co-crystallized ligand	PDB ID	RMSD (Ǻ)
CEL682	3LN1	1.7674
NPS5	3NT1	1.3330
DIF701	3N8Y	0.8436
IBP601	4PHA	1.0834
Molecule 8	3NT1	1.0810

**Fig. 11:**
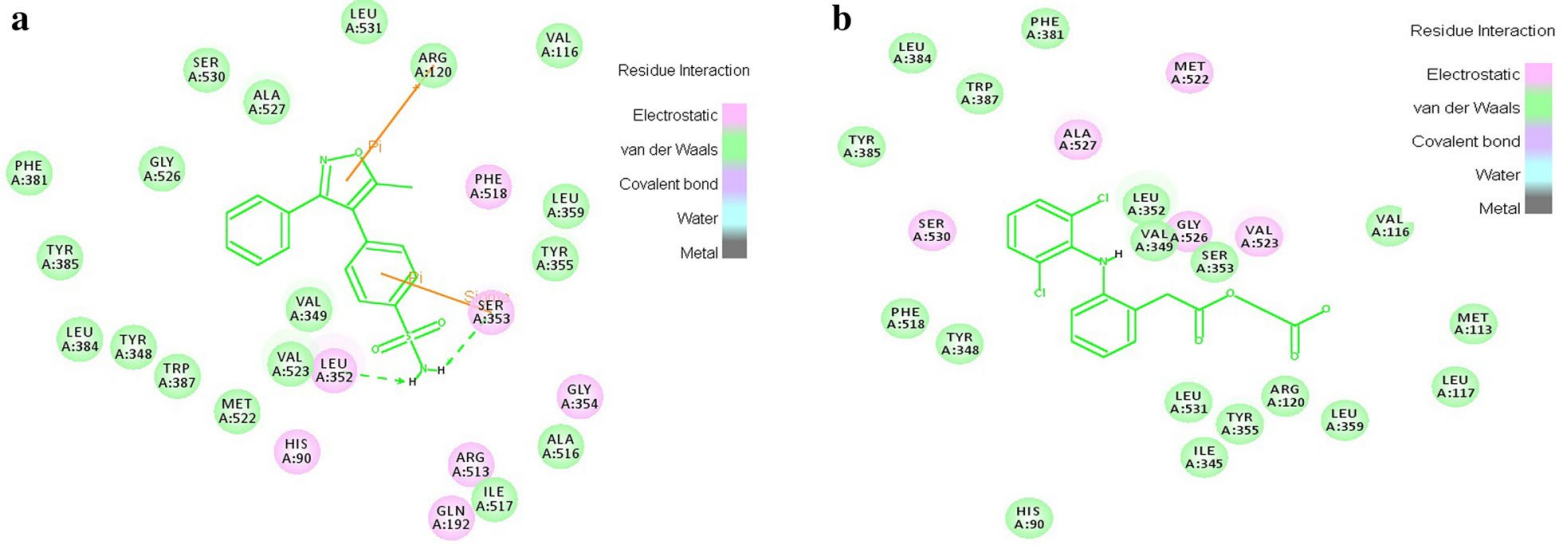
Interactions of selective and non-selective COX-2 inhibitors. **a** Rofecoxib **b** Aceclofenac

**Fig. 12:**
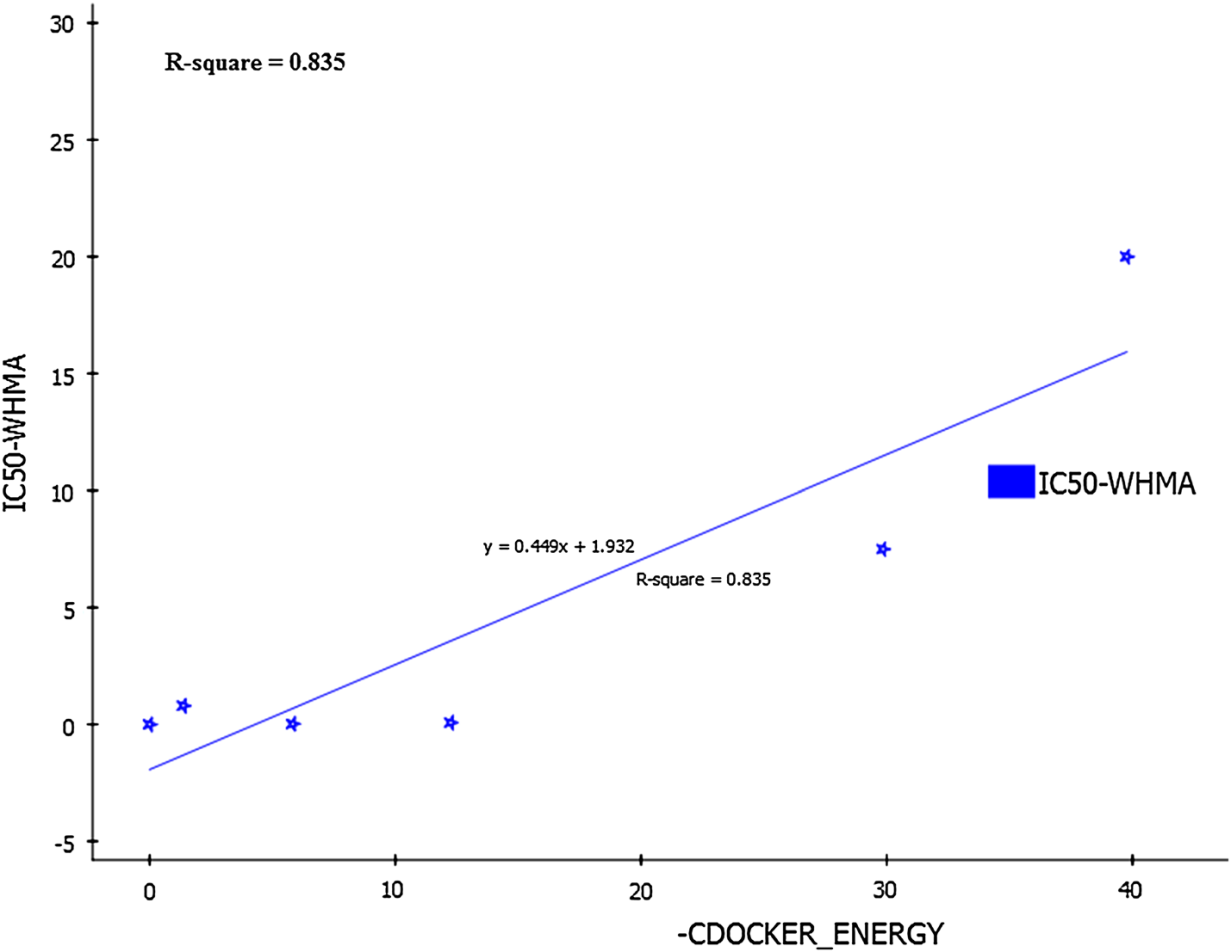
Correlation* point plot* of C-Docker energy and the experimental activity (IC_50_) of the nonselective COX-2 inhibitors

In the second method, the selected docking protocol parameters accurately distinguished the selective and non-selective COX-2 inhibitors. It is illuminated by the docking results in which C-Docker energy of selective COX-2 inhibitors fall in the negative kcal/mol range and the non-selective inhibitors energies fall in the range of positive kcal/mol (Table 7). Additionally, the binding site (3NT1) analysis of the drug receptor complexes revealed that all the selective COX-2 inhibitors formed π interaction with the active site amino acids which are major force for molecular recognition and join with hydrophobic interaction [22]. But, non-selective COX inhibitors formed hydrogen bond, VDW and electrostatic interactions only (Fig. 11). It clearly proves that the selective COX-2 inhibitors and designed 55 molecules possessed more selectivity compared to the non-selective inhibitors. This proposed model predicted the correlation between C-Docker energy and the experimental IC_50_ value of the selective and non-selective inhibitors. The correlation coefficient was predicted to be 0.835 (r^2^) (Fig. 12). This correlation strongly indicates that the docking protocol of this study possessed good predicting ability as well as it distinguishes the selective and non-selective COX-2 inhibitors precisely.

**Table 7:** C-Docker energy values of the selective and non-selective inhibitors

Selective COX-2 inhibitors	C-Docker energy value (kcal/mol)	Non-selective COX-2 inhibitors	C-Docker energy value (kcal/mol)
Rofecoxib	−19.0343	Diclofenac	5.45905
Valdecoxib	−9.2766	Ketorolac	12.2429
Etoricoxib	−3.32262	Aspirin	29.113
		Naproxen	32.0361
		Ibuprofen	39.7383

### Toxicity

#### ADMET descriptors

**Fig. 13:**
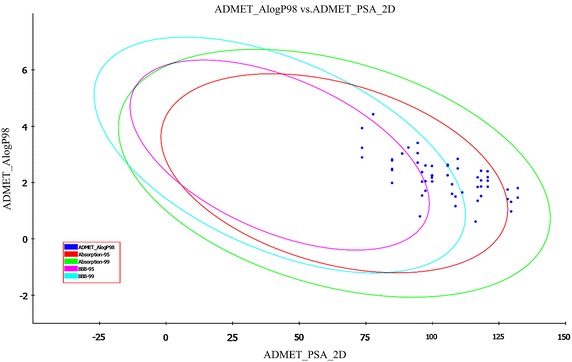
The 95 and 99 % confidence limit ellipses corresponding to the BBB and HIA models for tyrosine derivatives

In the present work, we have assessed ADMET (absorption, distribution, metabolism, excretion, and toxicity) properties of the 35 compounds which were selected from the docking report. ADMET descriptors were calculated to filter the poor tyrosine molecule with undesired pharmacokinetic and toxicity properties [23]. This step prevents wasting of time, chemicals as well as animal studies of tyrosine derivatives. The pharmacokinetic profile of all the molecules was predicted by means of six pre-calculated ADMET models provided by ADS 2.5 software. The ADMET plot shows the 95 and 99 % confidence ellipse for the HIA and BBB models (Fig. 13). The 95 % confidence ellipse represents the region of chemical space with molecules having excellent absorption through cell membrane. According to this model, for a designed molecule to have an optimal cell permeability, it should follow the criteria of PSA < 140 Å^2^ and AlogP98 < 5) [24]. The selected 35 molecules have shown PSA < 140 Å^2^ and AlogP98 < 5 which satisfied the criteria.

These selected molecules as well as standard celecoxib fall in the 95 and 99 % confidence ellipse for both HIA and BBB (Fig. 13). The HIA of the tyrosine derivatives ranges from 0 (good absorption) to 1 (moderate absorption) (Table 8). It indicates the good bioavailability of designed molecules to produce desired therapeutic effect. BBB penetration of the designed molecules indicated undefined to low penetration, except the molecule **141.** On the other hand, celecoxib exhibited moderate penetration to the BBB (Table 8). The aqueous solubility plays a vital role in the bioavailability of the drug. The designed tyrosine derivatives have solubility in the range of 2 (low soluble) to 3 (soluble) as referred in Table 9. Further, the hepatotoxicity level of all the molecules were calculated, the molecules with liver toxic nature were filtered out. Similarly, all the molecules were found to be satisfactory with respect to CYP 450 2D6 liver enzyme, suggesting that the tyrosine derivatives were non inhibitors of the metabolic enzyme. Finally, the PPB prediction denotes that all the designed molecules have binding ≤90 % clearly revealing that the molecules have good bioavailability and are not likely to be highly bound to carrier proteins in the blood [25].

**Table 8:** ADMET predictions of 35 tyrosine molecules and celecoxib

Name of the molecule	Absorption level	AlogP98	PSA 2D	BBB level	Solubility	Solubility level	Hepatotoxicity level	CYP 2D6	PPB level
Molecule_6	0	2.843	109.513	4	−4.495	2	0	0	0
Molecule_8	0	2.634	105.719	4	−4.197	2	0	0	0
Molecule_9	0	1.862	118.273	4	−3.523	3	1	0	0
Molecule_10	1	1.317	129.534	4	−3.217	3	0	0	0
Molecule_11	0	2.402	120.689	4	−4.027	2	0	0	0
Molecule_12	0	1.852	120.774	4	−3.883	3	1	0	0
Molecule_13	0	2.419	118.273	4	−4.009	2	1	0	0
Molecule_14	1	1.804	132.035	4	−4.096	2	0	0	0
Molecule_15	0	3.047	94.458	3	−4.388	2	1	0	0
Molecule_17	0	2.503	109.513	4	−4.197	2	1	0	0
Molecule_20	0	1.522	118.273	4	−3.225	3	0	0	0
Molecule_21	1	0.976	129.534	4	−2.919	3	0	0	0
Molecule_23	0	2.068	120.774	4	−4.071	2	0	0	0
Molecule_24	0	2.078	118.273	4	−3.711	3	0	0	0
Molecule_25	1	1.464	132.035	4	−3.798	3	0	0	0
Molecule_26	0	2.707	94.458	3	−4.09	2	1	0	0
Molecule_50	0	2.599	105.719	4	−3.984	3	0	0	0
Molecule_51	0	2.186	116.98	4	−3.793	3	0	0	0
Molecule_54	0	0.613	116.198	4	−2.789	3	0	0	0
Molecule_58	0	2.259	105.719	3	−3.686	3	0	0	0
Molecule_67	0	1.354	116.98	4	−2.83	3	0	0	0
Molecule_99	0	3.245	90.972	3	−4.639	2	1	0	0
Molecule_102	0	1.505	108.662	3	−3.598	3	1	0	0
Molecule_103	0	2.59	99.817	3	−4.351	2	1	0	0
Molecule_113	0	1.164	108.662	3	−3.3	3	1	0	0
Molecule_115	0	2.256	99.902	3	−4.114	2	1	0	0
Molecule_117	0	1.652	111.163	4	−3.925	3	1	0	0
Molecule_141	0	3.937	73.586	2	−4.973	2	1	1	0
Molecule_146	0	0.801	95.326	3	−2.859	3	1	0	0
Molecule_154	0	2.18	99.817	3	−3.996	3	1	0	0
Molecule_7	0	2.193	120.774	4	−4.182	2	0	0	0
Molecule_52	1	1.753	128.241	4	−3.584	3	1	0	0
Molecule_57	0	3.409	94.458	3	−4.521	2	0	0	0
Molecule_59	0	1.846	116.98	4	−3.495	3	0	0	0
Molecule_60	1	1.413	128.241	4	−3.286	3	1	0	0
Celecoxib	0	4.428	77.75	2	−6.603	1	1	0	1

**Table 9:** ADMET descriptor models

Name of the ADMET model	Prediction levels
Human intestinal absorption	0 (Good absorption)
	1 (Moderate absorption)
	2 (Low absorption)
	3 (Very low absorption)
Aqueous solubility	0 (Extremely low)
	1 (No, very low, but possible)
	2 (Yes, low)
	3 (Yes, good)
	4 (Yes, optimal)
	5 (Too soluble)
Blood brain barrier (BBB)	0 (Very high penetration)
	1 (High penetration)
	2 (Medium penetration)
	3 (Low penetration)
	4 (Undefined penetration)
Cytochrome P450 2D6 (CYP 2D6)	0 (Non−inhibitor)
	1 (Inhibitor)
Hepatotoxicity	0 (Nontoxic)
	1 (Toxic)
Plasma protein binding (PBB)	0 (Binding is <90 %)
	1 (Binding is >90 %)
	2 (Binding is >95 %

#### Osiris property explorer

The result of toxicity analysis of designed molecules showed low toxicity tendency except the molecules **103** and **113**. The drug-likeness value of standard and designed molecule exhibited the fragment content of the drug. If the drug-likeness value of designed molecules is increasing, then it has the same fragment content with existing drugs. Table 10 shows that the drug-likeness value of the tyrosine derivatives were higher than the standard celecoxib (−8.11), with the exception of **102**, **103**, **117**, **141**, **146** and **154** (−10.82 to −11.92). This results predict that among 35, 29 molecules exhibited same fragment content of the drugs. It confirms the drug likeness properties of these compounds.

**Table 10:** Toxicity of tyrosine derivatives and standard drug based on OSIRIS property explorer

Molecule	Mutagenicity	Tumorigenic	Irritant	Reproductive effect	Drug likeness	Drug score
Molecule_6	Green	Green	Green	Green	1.88	0.63
Molecule_8	Green	Green	Green	Green	2.25	0.62
Molecule_9	Green	Green	Green	Green	1.83	0.60
Molecule_10	Green	Green	Green	Green	2.46	0.66
Molecule_11	Green	Green	Green	Green	0.87	0.53
Molecule_12	Green	Green	Green	Green	2.46	0.67
Molecule_13	Green	Green	Green	Green	2.61	0.65
Molecule_14	Green	Green	Green	Green	−2.08	0.39
Molecule_15	Green	Green	Green	Green	2.03	0.54
Molecule_17	Green	Green	Green	Green	4.74	0.54
Molecule_20	Green	Green	Green	Green	4.69	0.50
Molecule_21	Green	Green	Green	Green	5.29	0.55
Molecule_23	Green	Green	Green	Green	5.54	0.57
Molecule_24	Green	Green	Green	Green	5.43	0.53
Molecule_25	Green	Green	Green	Green	0.74	0.48
Molecule_26	Green	Green	Green	Green	4.88	0.45
Molecule_50	Green	Green	Green	Green	2.34	0.45
Molecule_51	Green	Green	Green	Green	1.46	0.60
Molecule_54	Green	Green	Green	Green	1.77	0.59
Molecule_58	Green	Green	Green	Green	4.31	0.51
Molecule_67	Green	Green	Green	Green	2.39	0.46
Molecule_99	Green	Green	Green	Green	−0.06	0.46
Molecule_102	Green	Green	Green	Green	−10.82	0.39
Molecule_103	Green	Yellow	Red	Green	−15.1	0.18
Molecule_113	Green	Green	Red	Green	−7.79	0.32
Molecule_115	Green	Green	Green	Green	−7.28	0.33
Molecule_117	Green	Green	Green	Green	−11.92	0.33
Molecule_141	Green	Green	Green	Green	−17.18	0.34
Molecule_146	Green	Green	Green	Green	−11.29	0.35
Molecule_154	Green	Green	Green	Green	−8.91	0.31
Molecule_7	Green	Green	Green	Green	3.47	0.70
Molecule_52	Green	Green	Green	Green	−2.79	0.21
Molecule_57	Green	Green	Green	Green	−0.83	0.49
Molecule_59	Green	Green	Green	Green	4.36	0.52
Molecule_60	Green	Green	Green	Green	2.39	0.29
Celecoxib	Green	Green	Green	Green	−8.11	0.37

The drug score value is the combination of solubility, molecular weight, logP, drug likeness and toxicity risk. It is used for evaluating the potential of the drug candidate. When the drug score is better, then the compound is predictive to be a drug candidate [26]. The drug score value of standard celecoxib is found to contain 0.37. Finally 19 compounds which possessed drug score greater than the standard were shortlisted for further studies (Tables 11, 12).

**Table 11:**
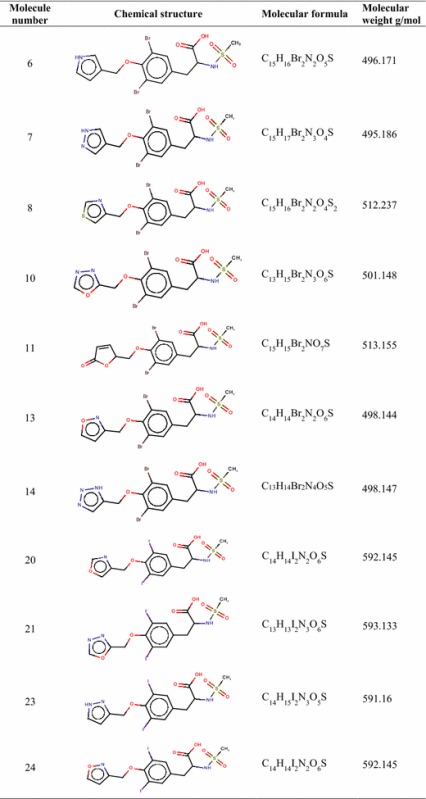
Details of shortlisted potent COX-2 inhibitors

**Table 12:**
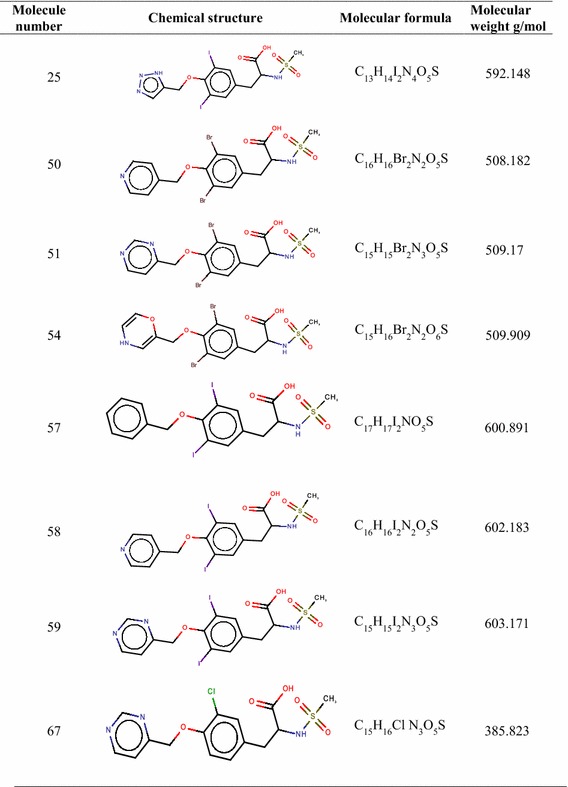
Details of shortlisted potent COX-2 inhibitors

## Conclusion

In the current work, 55 tyrosine structural analogues on docking with COX-2, COX-1 and hERG revealed that 35 molecules have more affinity at active site residues of COX-2 enzyme and less interaction with the other two proteins (COX-1, hERG) than standard celecoxib. This information proved to exhibit potential of high selective, less ulcerogenic and cardiotoxicity of the designed novel anti-inflammatory molecules. Further, the result of ADMET and Osiris property explorer helped to eliminate 16 unwanted toxic fragments contained tyrosine molecules. Finally, 19 hits with good pharmacokinetic parameter and negligible toxicity was proceeded for synthesis. Hence, it is concluded that the predicted parameters are exclusively used as a basis for the further design of tyrosine derivatives and understand the mechanism of COX-2 related enzymatic inhibition reactions. The next step of the potent safe anti-inflammatory drug identification involves the synthesis and biological evaluation of the selected molecules which are in progress.

## Abbreviations

ADS: accelyrs discovery studio
Arg: arginine
BBB: blood brain barrier
COX-1: cyclooxygenase-1
COX-2: cyclooxygenase-2
CYP 2D6: cytochrome P450 2D6
Gln: glutamine
HIA: human intestinal absorption
HM: homology modeling
His: histidine
Ile: isoleucine
Lue: leucine
PDB: Protein Data Bank
Phe: phenylalanine
PPB: plasma protein binding
PSA_2D: 2D polar surface area
RMS: root mean square
SAR: structure activity relationship
Ser: serine
TdP: torsade de pointes
Tyr: tyrosine
Val: valine
VDW: van der Waals
